# Insight From Animals Resistant to Prion Diseases: Deciphering the Genotype – Morphotype – Phenotype Code for the Prion Protein

**DOI:** 10.3389/fncel.2020.00254

**Published:** 2020-08-18

**Authors:** Ryan Myers, Alessandro Cembran, Pedro Fernandez-Funez

**Affiliations:** ^1^Department of Biomedical Sciences, University of Minnesota Medical School, Duluth, MN, United States; ^2^Department of Chemistry and Biochemistry, University of Minnesota Duluth, Duluth, MN, United States

**Keywords:** prion disease, prion protein, disease susceptibility, animal models, protein structure, structure–function, amino acid substitution

## Abstract

Prion diseases are a group of neurodegenerative diseases endemic in humans and several ruminants caused by the misfolding of native prion protein (PrP) into pathological conformations. Experimental work and the mad-cow epidemic of the 1980s exposed a wide spectrum of animal susceptibility to prion diseases, including a few highly resistant animals: horses, rabbits, pigs, and dogs/canids. The variable susceptibility to disease offers a unique opportunity to uncover the mechanisms governing PrP misfolding, neurotoxicity, and transmission. Previous work indicates that PrP-intrinsic differences (sequence) are the main contributors to disease susceptibility. Several residues have been cited as critical for encoding PrP conformational stability in prion-resistant animals, including D/E159 in dog, S167 in horse, and S174 in rabbit and pig PrP (all according to human numbering). These amino acids alter PrP properties in a variety of assays, but we still do not clearly understand the structural correlates of PrP toxicity. Additional insight can be extracted from comparative structural studies, followed by molecular dynamics simulations of selected mutations, and testing in manipulable animal models. Our working hypothesis is that protective amino acids generate more compact and stable structures in a C-terminal subdomain of the PrP globular domain. We will explore this idea in this review and identify subdomains within the globular domain that may hold the key to unravel how conformational stability and disease susceptibility are encoded in PrP.

## Introduction

The prion protein (PrP) is a 230 amino acid-long secreted glycoprotein anchored to the extracellular aspect of the membrane by a C-terminal glycosylphosphatidylinositol (GPI) anchor. PrP is highly expressed in brain neurons, but mice devoid of PrP (*Prnp0/0*) are viable and only show mild behavioral perturbations (Bueler et al., 1992; Tobler et al., 1996; Schmitz et al., 2014). PrP plays a central role in prion diseases in humans, a heterogeneous class of neurodegenerative disorders with cognitive, movement, or sleep manifestations (Zlotnik and Rennie, 1965; Mathiason, 2017). Prion diseases or transmissible spongiform encephalopathies (TSE) are fairly unique because they can present with sporadic, genetic, and infectious etiologies. The transmissible agent is proposed to be a proteinaceous molecule highly resistant to denaturing agents that contains misfolded conformations of PrP (resistant PrP [PrP^res^] or scrapie PrP [PrP^Sc^]) and other factors (Prusiner, 1998). Although rare, these are devastating diseases with an aggressive course of a few months from clinical manifestation and no effective treatments.

Another unique feature of these disorders is that they have direct pathological correlates in other animals, but are limited to some mammals. The common pathological features of human and animal TSEs are vacuolar (spongiform) degeneration of the brain and accumulation of misfolded, aggregated PrP conformations (Colby and Prusiner, 2011; Kraus et al., 2013; Scheckel and Aguzzi, 2018). Other than humans, some ruminants are the only mammals known to develop endemic prion diseases: scrapie in sheep and goat, and chronic wasting disease (CWD) in deer and moose (Mathiason, 2017). Several mammals proved susceptible to TSE in the laboratory in early transmission experiments following the discovery of kuru in the 1950s: chimpanzee, mouse, hamsters, bank vole (Chandler and Fisher, 1963; Zlotnik and Rennie, 1963, 1965; Chandler, 1971). Interestingly, one lab animal proved resistant to prions: the rabbit (Gibbs and Gajdusek, 1973; Barlow and Rennie, 1976). Decades later, a large-scale unintended experiment resulted in the zoonotic transmission of prions to cattle, which developed a new disease, bovine spongiform encephalopathy (BSE), or mad-cow disease, that was traced back to the consumption of scrapie-contaminated bone meal (Wells et al., 1987; Wilesmith, 1988; Winter et al., 1989). Shortly thereafter many domestic and zoo animals exposed to BSE-contaminated prions - felines, mustelids, and others – developed new prion diseases, expanding the TSE universe (Kirkwood and Cunningham, 1994; Sigurdson and Miller, 2003). Remarkably, a few animals exposed to the same contaminated feed seemed to be resistant to prion diseases: horse, domestic dog and other canids (wolf, coyote), and pigs (Kirkwood and Cunningham, 1994). The unfortunate spread of TSEs revealed a heterogeneous landscape of susceptibility to prion diseases, with some animals suffering endemic disease, others easily infected in the lab, and others showing a relatively high or complete resistance to infection. This scenario presents a unique opportunity to uncover the molecular mechanisms mediating disease transmission and neurodegeneration.

## Animals Resistant to Prion Disease: Intrinsic vs. Extrinsic Factors

Prion diseases affect humans and other mammals, but not birds or other vertebrates. The fact that distant mammals like humans and ungulates develop sporadic and infectious forms of TSEs may erroneously suggest that all mammals are equally susceptible to TSEs. Early studies on TSEs assumed that these conditions were caused by some type of small virus. The susceptibility to these new infectious agents was tested by inoculating brain homogenates from affected humans and sheep into several animals, including apes, New- and Old-World monkeys, rats, guinea pigs, cats, and rabbits (Gibbs and Gajdusek, 1973; Barlow and Rennie, 1976). These animals received intracerebral injections from kuru or Creutzfeldt-Jacob disease (CJD) human extracts or with ME7 scrapie from sheep. This intracerebral route accelerated the disease course and shortened the incubation time, maximizing the possibility of identifying positives by clinical or pathological analysis. The first set of experiments showed that human prions can be transmitted to apes, monkeys, and cats, but were unsuccessful in rabbits (Gibbs and Gajdusek, 1973). A few years later, ME7 scrapie was inoculated into the brains of rats, guinea pigs, and rabbits. Whereas rats demonstrated a pattern of disease progression similar to that seen in mice, guinea pigs and rabbits showed no disease, although guinea pigs showed low level prion replication (Barlow and Rennie, 1976). These two studies showed that prions did not replicate in rabbits and the infectious agent was quickly disposed of in rabbits.

Now that TSEs are well-characterized pathologically and molecularly, including the key role of PrP as the disease-causing agent, it is clear that few mammals suffer prion diseases under natural conditions, suggesting underlying differences in their susceptibility to TSE. The most significant animals lacking TSE are rabbits, horses, dogs, and pigs. Of these four, only one is a laboratory animal, the rabbit, and the rest are large and have long lifespans, making them unsuitable for experimental work. At this time, we only have positive or negative evidence for animals directly exposed to BSE during the mad-cow epidemics in the United Kingdom. Thus, animals not present in zoos nor fed the same contaminated bone meal could theoretically be susceptible or resistant within the known spectrum. Why is it important to understand the risk of TSE transmission for other animals? Because many domestic and wild animals are part of the human food chain and even those not eaten by humans may shed prions in the environment that could be transmitted to other animals. Additionally, studying animals naturally susceptible or resistant to TSE can contribute to decipher the molecular mechanisms governing the pathogenesis of TSEs. Despite the clear challenges of studying non-model animals, modern technologies provide the ability to study the structure and biological properties of PrP from many animals. These experiments can help better understand the mechanisms responsible for the spectrum of TSE susceptibility among mammals. Lastly, studying variations in a protein for many animals allows us to infer the evolutionary processes shaping PrP: natural selection or neutral genetic drift with unintended consequences in post-reproductive age.

The different animal susceptibility to TSEs led to two hypotheses to explain its underlying mechanisms: intrinsic factors (sequence-structure) vs. extrinsic factors (cellular milieu, cofactors) regulate TSE susceptibility. These two mechanisms cannot be separated when highly resistant animals (rabbits) are infected with prions. But PrP from these animals can be studied *in vitro*, *ex vivo*, and *in vivo* in the cellular context of susceptible animals and, vice versa, susceptible PrP can be studied in the cell context of resistant animals. Rabbit epithelial RK13 cells with low or undetectable levels of endogenous PrP transfected with ovine PrP (Rov9) result in high susceptibility to infection by sheep prions (Vilette et al., 2001). Moreover, transgenic rabbit expressing ovine PrP are susceptible to disease (Sarradin et al., 2015). This is evidence, along with other persuasive experiments, that rabbit cells do not express co-factors that inhibit prion replication, further supporting the idea that PrP conversion is mainly encoded by intrinsic factors. Thus, natural variations in the PrP sequence affecting its conformational dynamics is the likely mechanism underlying disease susceptibility. Hence, identifying the key residues conferring conformational stability/instability to the globular domain of PrP will contribute to uncover the molecular mechanisms mediating PrP neurotoxicity and disease susceptibility. However important, sequence is not the only intrinsic determinant of PrP aggregation dynamics and toxicity. Recent studies reveal a key contribution of post-translational modifications, particularly glycosylation, on the efficiency of PrP aggregation, fidelity of strain replication, neurotropism, and toxicity (reviewed in Baskakov et al., 2018). PrP contains two facultative *N*-glycosylation sites leading to three co-existing isoforms. Changes in PrP sequence can modulate the accessibility to the glycosylation sites whereas the ratio of the three resulting isoforms can restrict their possible quaternary assemblies due to the steric limitations imposed by the large glycans. Although glycosylation is a critical determinant of several properties of prions, we will focus this review on the impact of sequence variations in PrP conformational dynamics and toxicity.

## PrP 3D Structure: NMR and X-Ray Crystallography

**FIGURE 1:**
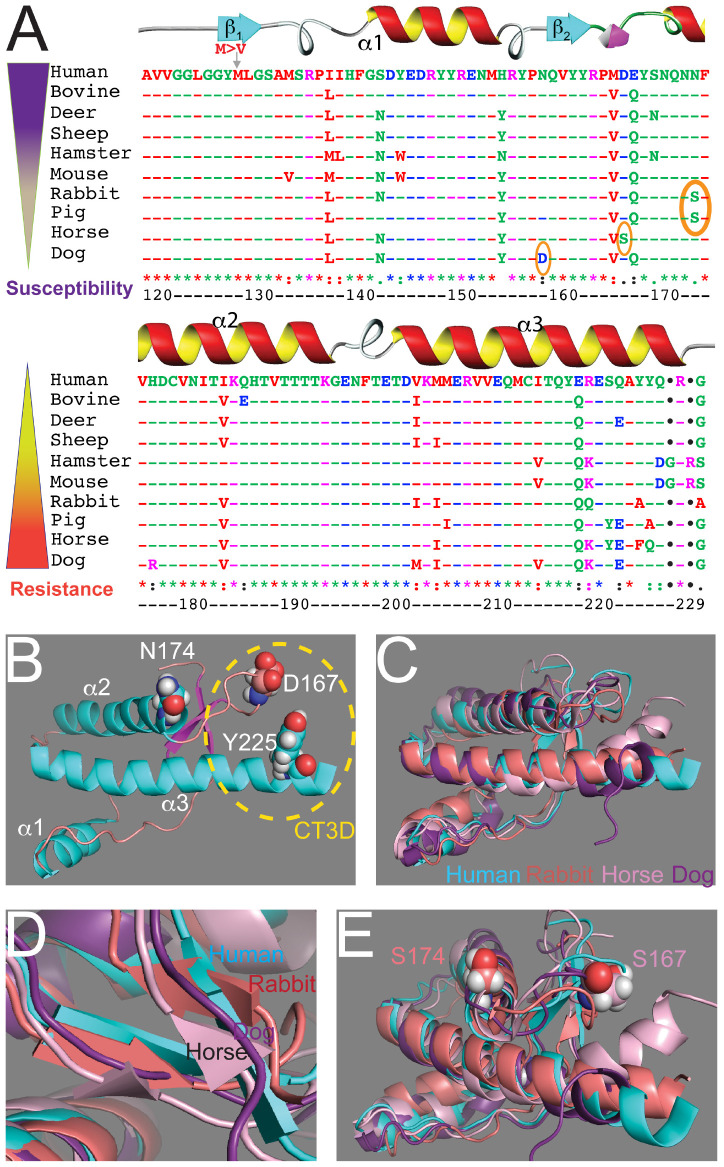
Sequence and structural alignments for the globular domains of mammalian PrP. **(A)** Sequence alignment for the structured domain of human, bovine, deer, sheep, Syrian hamster, mouse, rabbit, horse, dog, and pig PrP. *Amino acid numbering refers to human PrP*. The secondary structure is overlaid on top. The three key residues considered to be protective are circled. On the left, two inverse gradients indicate disease susceptibility and resistance. **(B)** Structure of the globular domain of human PrP (1qm2). Three amino acids are highlighted: D167, N174, and Y225. **(C**–**E)** Alignment of the globular domain of human (cyan), rabbit (salmon, 2fj3), horse (pink, 2ku4), and dog (purple, 1xyk) PrP. **(C)** Notice the overall similarity and the small differences around the CT3D domain. **(D)** The β-sheet content is smaller in rabbit and horse PrP and it disappears in dog PrP. **(E)** Detail of the CT3D domain showing the position of D167 in horse PrP and S174 in rabbit PrP. This figure contains published materials collated for illustrative purposes.

The interest on PrP as an infectious agent responsible for incurable neurodegenerative disorders led to significant work to uncover its structure. The classic method for resolving the 3D structure of biomolecules is X-ray crystallography due to its high spatial resolution, but the limiting step is the crystallization of the purified molecule. The first resolution of the PrP structure was obtained by NMR (nuclear magnetic resonance) using solution full-length and C-terminal domain from both mouse and Syrian hamster PrP (Riek et al., 1996, 1997; James et al., 1997; Liu et al., 1999). These studies revealed an unstructured N-terminal fragment (23–124) and a C-terminal globular domain (125–228) containing three α-helices and a short antiparallel β-sheet between helices 1 and 2. Throughout the paper we identify amino acids based on the human PrP sequence to avoid confusion (Figure 1A). The structure of the globular domain is highly conserved between mice and hamsters, and later structures for human, sheep, bovine PrP and others showed that this basic organization is highly conserved (Figure 1B) (Zahn et al., 2000; Knaus et al., 2001). Contemporaneous studies established that disease transmission and neurodegeneration were associated with a loss of helical content and an increase of β-sheet content (β-state) (Telling et al., 1995). These studies assigned a key role to a 3D domain in the C-terminal region consisting of the β2-α2 loop and distal helix 3, the C-terminal 3D (CT3D) domain (Figure 1B). Remarkably, this is a region of high sequence variability (Figure 1A) and the proposed binding site of a hypothetical protein (Protein-X) necessary for PrP conversion (Telling et al., 1995; Kaneko et al., 1997).

The structure of PrP from several resistant animals (dog, horse, rabbit, pig) were resolved during the 2010s to uncover how PrP toxicity and replication ability are encoded (Lysek et al., 2005; Khan et al., 2010; Perez et al., 2010; Wen et al., 2010). Essentially, these studies showed that the basic structure of PrP from these animals is very similar to that of animals susceptible to TSEs (Figure 1C). The globular domains contain three helices, but the β-sheet seems shorter in dog, horse, and rabbit PrP (Figure 1D). Detailed analysis of these structures identified a significant surface charge change in dog PrP due to the presence of D159 instead of the common N159 (Lysek et al., 2005) and increased organization of the β2-α2 loop in both horse and rabbit PrP (Figure 1E) (Khan et al., 2010; Perez et al., 2010; Wen et al., 2010). The stability of the β2-α2 loop is accompanied by increased contacts with the distal portion of helix 3 in horse and rabbit PrP, resulting in more stabilizing interactions within the CT3D domain (Khan et al., 2010; Perez et al., 2010; Wen et al., 2010). The X-ray crystal of rabbit PrP revealed a new feature not observed by NMR: a helix-capping domain at the start of helix 2 created by a double hydrogen bond (H-bond) between N171 and S174 (Khan et al., 2010). This structure is not observed in rabbit PrP-S174N, supporting the relevance of this finding. Interestingly, pig PrP carries the same S174 residue, whereas most mammals carry N174, suggesting a shared stabilizing domain with rabbit PrP. However, the NMR structure for pig PrP does not show the helix-capping domain (Lysek et al., 2005), making this domain uncertain. The new structural features in rabbit PrP offer a unique opportunity to examine genotype – morphotype – phenotype correlations, but the structural and phenotypic impact of other rabbit-specific substitutions needs to be considered as well. Intriguingly, compared to rabbit PrP, the reported structural changes in dog and horse PrP are subtle, suggesting that either small changes are sufficient or several subtle changes cooperate to stabilize PrP^C^ and delay or prevent disease.

## Conformational Dynamics of PrP Probed by Molecular Modeling

Thanks to its relatively small size and to the abundance of experimental structures, the globular domain of PrP has been the subject of profuse computational studies. The Daggett group showed that at low pH, the β-strand structures extend beyond the short domain to include the N-terminus and almost the entire β2-α2 loop (Alonso et al., 2001). Later, the same group (DeMarco and Daggett, 2004) built a protofibril model consistent with experimental data in which the extended β-sheet formed the interface between PrP monomers. Simulations performed by the Thirumalai group (Dima and Thirumalai, 2004) identified two main regions of instability in the protein: the second half of helix 2 and the C-terminus of helix 3 (residues 213–223). Other works focused instead on the fibril-forming capabilities of shorter peptide sequences of PrP (Kuwata et al., 2003; Collu et al., 2018; Zheng et al., 2018b) and on the stability of individual secondary structure domains (Camilloni et al., 2012). Simulations of the mouse PrP showed that the pathogenic mutation D178N associated with inherited CJD or fatal familial insomnia lowered the stability of the β-sheet (Barducci et al., 2005) and another group attempted to map the unfolding of the entire structured domain of PrP (Chamachi and Chakrabarty, 2017; Singh et al., 2017). Collectively, these contributions indicate that the PrP domain encompassing the N-terminus, the β-sheet, the β2-α2 loop, and the α-helix 3 C-terminus are regions of instability that may be prone to unfolding and to protein aggregation. The Caflisch group (Huang and Caflisch, 2015a, b) underlined the critical role of Y169, a highly conserved residue in mammalian PrP, in stabilizing the 3_10_ helical turn involving residues 165–168 within the β2-α2 loop. These findings were confirmed by Parrinello (Caldarulo et al., 2017) employing advanced sampling techniques.

Although most works focused on the protein, others pointed out the relevance of the water structure and dynamics in the stability of PrP (De Simone et al., 2005) and in the formation of oligomers (Thirumalai et al., 2012). Other research has shown that the correct modeling of electrostatic interactions (Zuegg and Gready, 1999) is important to correctly describe the protein’s stability, which is also affected by histidine protonation states (Langella et al., 2004) and by pH (Campos et al., 2010). An endeavor to systematically characterize the differences in the secondary structure and in the flexibility of the protein for a large number of PrP species through molecular dynamics simulations was attempted by Zhang (2018). These studies identified a salt bridge between R164 and D178 (Zhang and Wang, 2016) as important for the β2-α2 loop stability.

## Purified and *in vitro* Models of Resistant PrP Misfolding

Until the early 2000s, the evidence accumulated from highly resistant animals consisted of laboratory experiments with rabbits and negative epidemiological data for non-model animals: horses, dogs and other canids, and pigs. Modern biological and biochemical techniques enable the study of the intrinsic properties of PrP from these animals: *in vitro*, *ex vivo*, and transgenic animals. At the most basic level (sequence), the key question is determining which amino acid changes are responsible for altering PrP biological properties. At a deeper level, the idea is to understand how specific amino acid changes impact PrP conformation and dynamics by either enhancing or suppressing PrP misfolding, propagation, and toxicity. This key insight will shed light on the elusive genotype – morphotype – phenotype correlation.

The focus on rabbit PrP brought forth a number of studies to uncover the mechanisms mediating resistance to TSE. Though over a decade apart, two studies emerged as highly similar in goals (Vorberg et al., 2003; Eraña et al., 2017). These studies identified 22 amino acid differences between full-length rabbit and mouse PrP, and set out to determine which residues impact prion transmissibility by complementary approaches. These studies offer a unique opportunity to analyze the impact of complementary amino acid substitutions in either the mouse or rabbit PrP backbones. First, the Priola group used a scrapie-infected mouse neuroblastoma (Sc^+^-MNB) cell model persistently infected with a mouse-adapted scrapie prion (RML) (Vorberg et al., 2003). The Sc^+^-MNB cells express WT and recombinant mouse PrP (recPrP) carrying rabbit PrP-specific amino acid changes. The assay consisted on determining which substitutions inhibited the ability of recPrP from replicating prions. More recently, the Castilla group used recombinant rabbit PrP carrying mouse-specific changes (Eraña et al., 2017). Their approach was to use the powerful cell-free PMCA (protein misfolding cyclic amplification) technique (Saborio et al., 2001) to determine which mouse-specific substitutions enabled conversion of the naturally resistant rabbit PrP.

**FIGURE 2:**
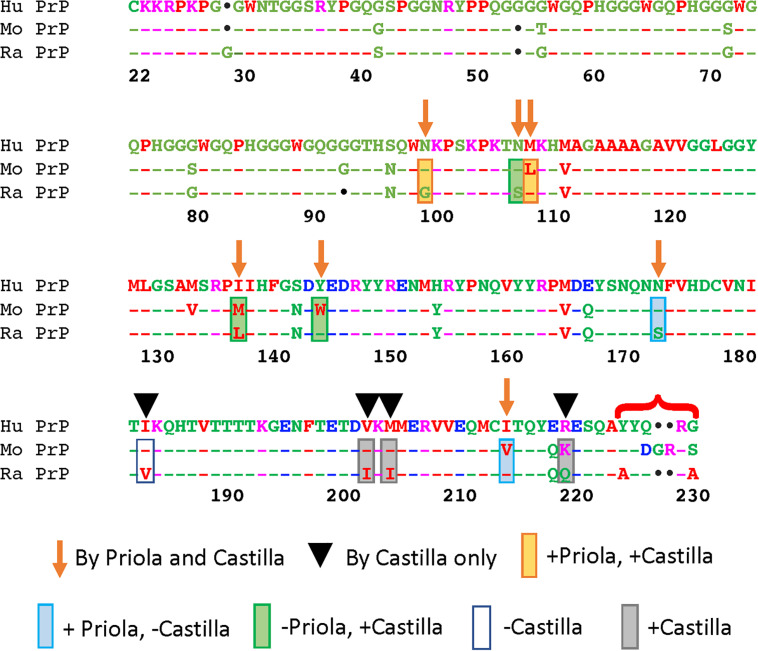
Functional consequences of complementary amino substitutions in mouse and rabbit PrP. Sequence alignment of human, mouse, and rabbit PrP highlighting the amino acids that were mutated in mouse PrP (arrows) (Vorberg et al., 2003) and those introduced in rabbit (arrows and arrowheads) (Eraña et al., 2017). The bracket indicates differences in the C-terminal that were not modified in these studies. Orange boxes: amino acid changes that worked in both assays. Blue boxes: amino acid changes that worked in mouse but not in rabbit. Green boxes: amino acid changes that worked in rabbit but not in mouse. Gray boxes: amino acid changes that worked in rabbit. Blank box: amino acid change that did not work in rabbit. This figure contains published materials collated for illustrative purposes.

Of the 22 differences between mouse and rabbit PrP (Figure 2), six reside in the unstructured N-terminal domain. Previous studies have shown that residues 1–94 do not play any significant roles in TSE resistance (Rogers et al., 1993; Fischer et al., 1996; Lawson et al., 2001). Of the 16 remaining, Priola introduced seven substitutions into mouse PrP, whereas Castilla introduced the same seven plus an additional four located in the C-terminal region into rabbit PrP (Figure 2) (Vorberg et al., 2003; Eraña et al., 2017). Of notice, both studies skipped the five amino acid differences at the end of the C-terminal (Figure 2) in part because their proximity to the GPI anchor makes them less likely to contribute to the mechanism of misfolding. For Priola, four of the seven residue replacements inhibited mouse PrP^res^. For Castilla, 8 of the 11 replacements enabled conversion of rabbit PrP. These complementary studies agreed on two effective replacements: N/G100 and L/M109. The studies describe conflicting results for five residues, where the changes affected one assay but not the other: N/S108, M/L138, Y/W145, N/S174 and I/V215 (Vorberg et al., 2003; Eraña et al., 2017). The Castilla group further tested the eight protective residues in rabbit PrP with a new prion strain, but only three permitted conversion this time: N/S108, M/L109, and V/I203. Moreover, this group validated their observations by introducing 11 amino acid replacements from rabbit on mouse PrP. Unexpectedly, all 11 changes decreased the propagation activity below WT, with N100G completely inhibiting propagation (Eraña et al., 2017). Interestingly, both groups created the corresponding double mutants N/S108 and M/L109 in the mouse and rabbit backbones. Both experiments were negative, reversing the positive effect of the single mutants. These studies highlight the asymmetric impact of the reverse amino acid substitutions on rabbit and mouse PrP, and lack of cooperativity of effective substitutions, underscoring our limited understanding of the rules governing PrP conformational dynamics.

Since the discovery that TSEs are caused by the conversion of endogenous PrP from a primarily α-helical state into a mostly β-sheet state (β-state) (Swietnicki et al., 1997; Baskakov et al., 2001), extensive work has been invested to study the misfolding propensity of PrP from different animals. Chakrabartty’s group developed a method to test the structural state of recombinant PrP using circular dichroism to measure the propensity of PrP from various organisms to populate the β-state under favorable conditions for PrP misfolding like low pH (Khan et al., 2010). This group found that rabbit PrP had a much lower tendency to occupy the β-state than did hamster and mice PrP. Horse and dog PrP were even less likely to occupy the β-state than rabbit PrP, with dog PrP being the most resistant (Khan et al., 2010). This resistance changed for rabbit PrP-S174N, which correlates with the structural studies pointing to the key role of S174 in the formation of the helix-capping domain. These results add evidence to the stability of PrP from these animals and their resistance to misfolding critical for prion diseases.

## Highly-Resistant to Prion Disease, Not Impervious

With the use of PMCA, researchers have explored the limits of the resistance of rabbit, horse, and dog PrP. Recent experiments showed that rabbit PrP can replicate *in vitro* and that rabbits are susceptible to TSEs under highly favorable conditions. Using PMCA with various prion strains as seeds, they showed that rabbit PrP can be converted into PrP^res^ (Chianini et al., 2012). Even unseeded rabbit PrP^C^ was able to generate PK-resistant PrP through several rounds of PMCA, indicative of spontaneous conversion. It took at least three rounds of seeded PMCA to accomplish conversion of rabbit PrP; in the case of unseeded PrP, it took 13 rounds. When the novel rabbit PrP^res^ was inoculated into three rabbits expressing WT PrP, one of the rabbits developed prion disease. Upon second passage from this positive animal, two out of 10 rabbits developed disease and accumulated PrP^res^ (Chianini et al., 2012). These experiments demonstrate that rabbits are not absolutely resistant to prions, but they also underscore the difficulty of transmitting prions under highly favorable experimental conditions that are unlikely to be found in nature. Overall, compared to other animals, rabbits demonstrate a high degree of resistance to prion disease, a quality that is mainly encoded in its unique sequence and structure.

Pigs have received less attention than other resistant animals for two main reasons: they are large animals and no unique structural features were identified in PrP by NMR despite sharing the S174 substitution with rabbit PrP. Prior to the spread of the mad cow epidemic to other animals, experimental study of pigs was of little concern. Still, when they were challenged with a strain of Kuru, they remained resistant (Dawson et al., 1994; Jahns et al., 2006). When BSE came onto the scene, the concerns for transmission grew because pigs are not only a human food source but are also routinely fed scraps, including scrapie-infected scraps at that time. Parenteral inoculations of pigs with BSE successfully infected pigs, though they remained resistant to the oral route of infection (Dawson et al., 1990; Ryder et al., 2000; Wells et al., 2003; Konold et al., 2009). Despite confirmed experimental transmission of TSE, there remains no documented natural cases. Yet, because pigs for consumption are typically slaughtered at around 6 months of age, development of clinical TSE in pigs is unlikely because of the long-incubation times. However, some pigs are aged and used for breeding and still there are no observations of natural occurrence in that population, supporting a natural resistance to prions.

## Transgenic Animals Expressing Highly-Resistant PrP

The *in vitro* and *in vivo* studies reviewed above support the hypothesis that PrP conversion and TSE susceptibility are primarily or solely dependent on intrinsic factors encoding the conformational stability of PrP. However informative, *in vitro* studies indirectly infer the pathological consequences of the substitutions introduced on PrP. The next level is to study PrP from TSE-resistant animals and their protective residues in flexible animal models. The two genetic models used in the study of PrP are fruit flies (*Drosophila melanogaster*) and mice. Fruit flies are a powerful research tool that lacks endogenous PrP, which is not conserved in invertebrates, providing an ideal environment to study PrP behavior in a naïve system. Moreover, generating transgenic flies is economic and fast, enabling the generation of multiple transgenes (Pfeiffer et al., 2010; Mohr et al., 2014). Flies have a small but complex tripartite brain homologous to the mammalian brain (Reichert, 2005) that contains 10^5^ neurons, an estimated 10^6^ synapses, and well characterized centers that control sophisticated behaviors, providing a robust system for studying neurodegenerative diseases (Simpson, 2009; Bellen et al., 2010; Zheng et al., 2018a). Mice are more time-consuming and expensive but offer the best context for observing the disease process in a mammal. Additionally, mice lacking PrP (*Prnp0/0*) provide an empty genetic background to express heterologous PrP from other animals (Bueler et al., 1992).

Transgenic flies expressing PrP from Syrian hamster, mouse, or sheep show progressive neurodegenerative changes accompanied by PrP misfolding into relevant toxic conformations (Gavin et al., 2006; Fernandez-Funez et al., 2009; Thackray et al., 2012). Additionally, flies demonstrate high sensitivity to natural PrP sequence, resulting in a gradient of toxicity: hamster > mouse > rabbit (Fernandez-Funez et al., 2010). In support of this observation, we described a similar gradient in PrP misfolding and aggregation as demonstrated by sucrose gradient. In a follow-up study, we generated transgenic flies expressing WT rabbit, dog, and horse PrP. Neither PrP caused neurodegeneration confirming the hypothesized conformational stability of these disease-resistant PrPs (Sanchez-Garcia and Fernandez-Funez, 2018). On the other extreme of this continuum of PrP toxicity, expression of human PrP in flies leads to extremely high toxicity, including a new eye phenotype (Fernandez-Funez et al., 2017). Overall, these experiments support that the spectrum of PrP toxicity is due to changes in PrP sequence.

The Castilla group first used PMCA to produce PrP^res^ from rabbit and dog PrP with BSE as seed, resulting in “adapted” prion strains after several rounds: BSE-rabbit PrP^res^ and BSE-dog PrP^res^. These strains were then inoculated into bovine-PrP mice. First passage showed similar incubation time as cattle BSE-inoculated mice. Upon second passage, BSE-dog PrP^res^ and BSE-rabbit PrP^res^ had significantly reduced incubation times. In a different test against mice expressing human PrP, only BSE-rabbit established infection upon first passage (Vidal et al., 2013a). Often when a prion infects a new species, the incubation period is long and infectivity is low (Vidal et al., 2013b). However, once adapted to the new host, the incubation period is reduced and infectivity increases. This concern was addressed above by incorporating a second passage. The Castilla group further tested the susceptibility of rabbit PrP in transgenic mice. Intracerebral inoculation of transgenic mice overexpressing rabbit PrP with misfolded PrP seeds achieved 100% transmission (Vidal et al., 2015), a more efficient result than in wild type rabbits likely due to the overexpression of rabbit PrP. In an *in vitro* assay against several prion strains to demonstrate strain-specific susceptibility, rabbit PrP converted to PrP^Sc^ in all cases (Vidal et al., 2015). The story was different, though, in an *in vivo* test of those same strains against rabbit PrP in transgenic mice. Some of the strains induced infection to varying degrees, but others did not, including CWD and SSBP/1 (Vidal et al., 2015).

Another group generated transgenic mice expressing WT horse PrP (tgEq) at twice the levels that it is expressed in the horse brain (Bian et al., 2017). These mice were intracerebrally inoculated with various prion strains and observed for development of prion disease. Out of ten different strains, only strain SSBP/1 caused disease in tgEq PrP mice, albeit in only two of the six mice. Interestingly, upon second passage with brain homogenates from the infected mice into tgEq PrP mice, there was neither disease nor accumulation of PrP^Sc^ (Bian et al., 2017). However, transgenic mice expressing ovine PrP did develop symptoms when inoculated with the same homogenate from SSBP/1-tgEq PrP mice. In another part of this study, PMCA was used to convert horse PrP^C^ into PrP^Sc^, but when it was inoculated into tgEq PrP mice, the mice did not develop pathology. This report strongly supports the stability of horse PrP and high resistance to misfolding and TSE infection, although a small number of cases were positive. Considering that these tgEq PrP are inoculated by a non-natural route (intracerebral) that directly exposes PrP^C^ to the inoculum, horse PrP demonstrates high resistance to conversion described in *in vitro* (Khan et al., 2010) and *Drosophila* models (Sanchez-Garcia and Fernandez-Funez, 2018).

As with horse and rabbit PrP, researchers forced dog PrP^C^ to convert to PrP^Sc^. To test the stability of dog PrP, they once again utilized the powerful PMCA to induce the misfolding of dog PrP in a cell-free system (Fernandez-Borges et al., 2017). Dog PrP proved to be incredibly resistant, but after several rounds of PMCA, they were able to generate PrP^Sc^ only with two of the six prion strains used as seeds: classical BSE and sheep BSE.

Pigs are critical in the human food chain and understanding the risk of contracting prion diseases from other animals is a key economic issue. Generation of transgenic mice expressing pig PrP allowed for quicker experimental studies of pig PrP outside of its normal host. The Torres group introduced pig PrP into *Prnp0/0* mice (poTg001) in which pig PrP was expressed fourfold in the mouse brain than in the pig brain (Castilla et al., 2004). The poTg001 model was used in successive studies challenging the pig PrP with an array of prion strains (Castilla et al., 2004; Espinosa et al., 2009, 2020). Only classic BSE and some BSE-derived strains successfully infected the mice, but with a low attack rate. The studies report conflicting results on the infectivity of one scrapie strain: in one study it caused conversion – at a low attack rate – while in a later study it did not cause PrP conversion. Similar results were observed in *in vivo* experiments using PMCA with the panel of prion strains (Espinosa et al., 2020).

## Protective Activity of Unique Residues From Resistant Animals

After reviewing the evidence supporting the different susceptibility of animals to TSEs, the main hypothesis is that amino acid changes on PrP encode its conformational dynamics and propensity to cause disease. The next step is to determine *how* specific amino acids induce conformational changes that result in high vs. low toxicity in animal models. Fortunately, many PrP sequences and structures are available, providing unparalleled resources for addressing this critical question. The most N-terminal domain (residues 1–94) does not appear to drive PrP conversion (Rogers et al., 1993; Fischer et al., 1996; Lawson et al., 2001). Therefore, we will focus mainly on the globular domain. Although the overall PrP sequence and structure are highly conserved in mammals, several changes are evident (Figures 1A,C). Sequence alignments identify 10–15 amino acid changes between human and other animals only in the globular domain. Of these, many are conservative changes not expected to largely impact the 3D conformation. The alignment shows relatively high variation in the β2-α2 loop (residues 166–170) and in the C-terminus of helix 3 (residues 219–229). Interestingly, these two regions are spatially close and several contacts are confirmed by structural studies, indicating that the β2-α2 loop and the C-terminus of helix 3 form a 3D domain. The variability in the distal helix 3 has been traditionally assumed to have less impact on the globular domain because of its proximity to the GPI anchor, which may underestimate the role of these variants. Combining sequence and structural data revealed three prominent amino acid changes likely to encode PrP conformational stability: D159 in dog, S167 in horse, and S174 in rabbit and pig. Next, we will review the work done in transgenic animals to examine the consequences of altering these residues.

## Dog PrP – D159

Most animals, including humans, have an asparagine (N) at position 159, but dogs and other members of the Canidae family (wolf, fox, coyote) have either an aspartic acid (D) or a glutamic acid (E) at this position. Two mustelids, the wolverine and the marten, also share this acidic residue at 159 (Stewart et al., 2012; Fernandez-Borges et al., 2017). The NMR structure of dog PrP shows a conserved globular domain with subtle changes. The short β-sheet seems to be gone by NMR and the surface charge is more negative around D159 due to its negative charge and the increased solvent exposure (Lysek et al., 2005). This change in the surface charge may affect the interactions with other proteins like chaperones. Utilizing the versatile fruit fly, we generated transgenic flies expressing mouse PrP with the N159D substitution (Sanchez-Garcia et al., 2016). Expression of mouse PrP-N159D showed improved locomotor performance compared to flies expressing mouse PrP-WT, a change that correlated with lower levels of pathogenic conformations of PrP (Sanchez-Garcia et al., 2016). We recently conducted the reverse experiment by introducing the D159N substitution in dog PrP in flies. Flies expressing dog PrP-WT show no toxicity in behavioral and anatomical assays. However, flies expressing dog PrP-D159N exhibit progressive locomotor disfunction and degeneration of brain neurons (Sanchez-Garcia and Fernandez-Funez, 2018). The consistent results in the reverse substitutions in mouse and dog PrP strongly support the critical role of D159 in encoding higher PrP stability.

In continuation of their *in vitro* studies, the Castilla group generated transgenic mice expressing mouse PrP carrying the N159D substitution (Fernandez-Borges et al., 2017). When these mice were inoculated with prions, they showed no clinical signs nor accumulated PrP^Sc^. In a follow-up study, they found that co-expression of mouse PrP-N159D with mouse PrP-WT significantly increased survival, indicating that N159D has a dominant-negative effect on the ability of PrP-WT to misfold and induce disease (Otero et al., 2018). They next generated mice expressing bank vole PrP carrying the same N159D substitution with a polymorphism at residue 109 (I109) that further increases the propensity of bank vole PrP to spontaneously misfold (Otero et al., 2019). Challenging the mice expressing bank vole PrP-N159D with two prion strains resulted in a 100% attack rate, but the disease onset was significantly delayed compared to mice expressing a control bank vole PrP construct. A more recent study compared transgenic mice expressing dog PrP-WT and -D159N (Vidal et al., 2020). Of note, dog PrP-WT carried the E159 polymorphism instead of the typical D159. Mice expressing dog PrP-WT[E159] and -D159N were challenged with various prion strains: no prion strain propagated in dog PrP-WT[E159], but did so in the D159N model. Overall, experiments conducted in transgenic animals confirmed the protective activity of D/E159 in the context of dog, mouse, or bank vole PrP.

## Horse PrP – S167

The NMR structure of horse PrP revealed increased structural definition of the β2-α2 loop compared to mouse PrP (Perez et al., 2010). The NMR structure of mouse PrP carrying the horse substitutions D167S, Q168E, and N173K, along with double mutants, showed that D167S conferred the β2-α2 loop a well-defined structure and increased the long-distance interactions between the loop and helix 3, similar to those observed in horse PrP (Perez et al., 2010). Transgenic mice expressing high levels of mouse PrP-D167S developed spontaneous spongiform pathology, neurologic disease, and PrP^Sc^ deposits, whereas a control line overexpressing mouse PrP-WT (tga20) did not (Sigurdson et al., 2011). In contrast, mice expressing moderate levels of mouse PrP-D167S were similar to control mice, except for a lower fraction of insoluble PrP. The reverse experiment was conducted in transgenic flies expressing horse PrP-S167D. In contrast to flies expressing horse PrP-WT, expression of horse PrP-S167D showed aggressive locomotor dysfunction and degeneration of brain neurons (Sanchez-Garcia and Fernandez-Funez, 2018). Remarkably, horse PrP-S167D induced a form of neurodegeneration not seen before with other PrP in which the cell bodies swelled up causing a significant enlargement of the neuronal clusters (Sanchez-Garcia and Fernandez-Funez, 2018). The same cellular phenotype was described in flies expressing Aβ42 and linked to aberrant autophagy (Ling et al., 2009). So far, these limited studies show conflicting results regarding the protective activity of S167.

## Rabbit PrP – S174

S174 has been proposed as a key residue mediating the stability of rabbit PrP based on structural, biochemical, cell culture, and cell-free evidence (Vorberg et al., 2003; Khan et al., 2010; Wen et al., 2010). The S174N substitution in rabbit PrP disrupted stability, changed overall surface charge, and made the β2-α2 loop less rigid and more flexible. Following on these studies, we generated transgenic flies expressing rabbit PrP-S174N expecting to find an increase in toxicity. However, flies expressing rabbit PrP-S174N in brain neurons exhibited no changes in locomotion nor in brain architecture (Sanchez-Garcia and Fernandez-Funez, 2018). These experiments were conducted in parallel with the D159N and S167D mutants in dog and horse PrP, respectively, that increased PrP toxicity. This puzzling result suggests that this single amino acid change has a limited impact on PrP structural dynamics *in vivo* inconsistent impact of S/N174 on *in vitro* PrP replication were reviewed above (Figure 2) (Vorberg et al., 2003; Eraña et al., 2017). Taken together, these results suggest that multiple amino acids contribute to the high stability of rabbit PrP and that no single amino acid is sufficient to induce dramatic changes on PrP. These observations still leave open the question about how is the conformational stability of rabbit PrP encoded in its sequence and structure. It is likely that multiple amino acids in the β2-α2 loop and helix 3 cooperate to increase the stability of the CT3D domain, complicating the experimental demonstration.

## Lessons Learned: How Is Disease Susceptibility Encoded in PrP Structure?

The evidence discussed so far agrees that rabbit, horse, dog, and pig PrP are comparatively more resistant to conversion and less toxic than PrP from naturally susceptible animals. However, under the right experimental conditions, rabbit, horse, dog, and pig PrP can convert into PrP^Sc^ and cause disease *in vitro* and *in vivo*. *In vitro* systems like PMCA provide a highly flexible environment that accelerates conversion by exploring high energy states perhaps facilitated by the inhibition of protective proteostasis mechanisms in cell-free systems. Conversion of rabbit PrP in PMCA resulted in prions that infected wild type rabbits, albeit with low efficiency, demonstrating the power of PMCA to lower the species and strain barriers (Fernandez-Borges et al., 2012). Mice expressing horse PrP seem to be permissive for spontaneous prionopathy, whereas mice expressing horse, rabbit, dog, and pig PrP were permissive to transmission of some prion strains. However, this experimental work does not mean that rabbits, dogs, horses, and pigs are naturally susceptible to TSE: these animals do not develop spontaneous disease. In fact, the experiments showed that these four PrPs are harder to convert than PrP from naturally susceptible animals like mouse or bank vole. Generating misfolded rabbit PrP required several rounds of PMCA (Vidal et al., 2015); horse PrP needed 14 rounds of PMCA (Bian et al., 2017) and dog PrP required a modified protocol for PMCA because 10 rounds of standard PMCA drew negative results (Fernandez-Borges et al., 2017). A previous report described that using more than one round of PMCA exceeds a natural test of the transmission barrier (Fernandez-Borges et al., 2009). Furthermore, the transmission studies were conducted by intracerebral inoculation, an unnatural and favorable mode of infection that skips the less efficient peripheral replication. It must be noted that transgenic mouse models overexpress rabbit, horse, and pig PrP^C^ multiple times the levels of endogenous PrP (Vidal et al., 2015), a condition that may further increase the likelihood of the mice developing spontaneous TSE. Overall, these studies demonstrate four important things. (1) The PrP from rabbits, dogs, horses, and pigs exhibit a remarkable resistance to conversion. (2) PMCA is a powerful tool to overcome the conversion-barrier. (3) PrP from these resistant animals can indeed misfold, in other words, they are not impervious to conversion. Thus, it is unlikely that any animal is completely resistant to forming PrP^Sc^ given the evolutionary constraints on PrP. (4) Although each of these four highly resistant PrPs show low susceptibility to misfolding, horse and dog PrP seem to be more resistant than rabbit and pig PrP under similar conditions, identifying two targets for further research.

The impact of D/E159, S167, and S174 on PrP structure has been tested in multiple systems (Table 1); of these three, S174 is the only proposed to generate a new structural feature. Unfortunately, the evidence for the protective activity of S174 is mixed: in some experiments, S/N174 modifications had the expected effect and in others, it did not (Vorberg et al., 2003; Khan et al., 2010; Eraña et al., 2017; Sanchez-Garcia and Fernandez-Funez, 2018). In contrast, D/E159 and S167 are associated with subtle structural changes and the experimental evidence is generally supportive for D/E159 being protective but is mixed for S167. The contradictory results observed in different assays add some confusion to the role of these three residues but are not completely surprising. The hypothesis is that amino acids with a determining protective role on PrP structure will show opposite effects when eliminated from a resistant PrP or introduced into a susceptible PrP. The lack of consistent results for the S/N174 manipulations suggest a relatively modest role, possibly in combination with other rabbit-specific amino acids. The strong effects observed on structural/biochemistry experiments are consistent with the ability to modify protein structures when the environment is controlled chemically (pH, urea). The most comparable experiments altering multiple amino acids in mouse and rabbit PrP are indeed quite different, one using cell culture and the other a cell-free system (PMCA) (Vorberg et al., 2003; Eraña et al., 2017). The cell-free system is expected to provide more flexibility by being subject to incubations and sonications, whereas living cells provide a more restrictive environment regulated by homeostatic mechanism. Rabbit PrP-S174N expressed in flies behaves the same as WT, supporting the buffering effect of the cellular environment (extrinsic factors) in response to mild structural changes. Overall, these results hint at a gradient of effects on PrP stability, with S174 having mild effects, followed by S167, and D/E159 having the most drastic protective effects despite the lack of novel structural perturbations.

**TABLE 1:** Summary of experimental manipulations of candidate protective residues 159, 167, and 174.

Residue	Assay/model	Result	Source
D/E/N159	NMR/X-ray of dog PrP	Change in area surface charge – more negative	Lysek et al., 2005
D/E/N159	Fruit flies expressing mouse PrP-N159D	N159D reduced the amount of PrPSc-like conformations	Sanchez-Garcia et al., 2016
D/E/N159	Fruit flies expressing dog PrP-D159N	D159N caused degeneration of brain neurons	Sanchez-Garcia and Fernandez-Funez, 2018
D/E/N159	Mice expressing mouse PrP-N159D	N159D was protective against TSE	Fernandez-Borges et al., 2017
D/E/N159	Mice co-expressing mouse PrP-WT and mouse PrP-N159D	Co-expression increased survival	Otero et al., 2018
D/E/N159	Mice expressing bank vole PrP-N159D	Disease onset was delayed	Otero et al., 2019
D/E/N159	Mice expressing dog PrP-D159N vs. D159E (WT polymorphism)	Prion strains were able to propagate in dog PrP-D159N, but not in dog PrP-WT	Vidal et al., 2020
S/D167	NMR on mouse PrP-D167S	β2-α2 loop more organized than mouse PrP-WT	Perez et al., 2010
S/D167	Fruit flies expressing horse PrP-S167D	S167D caused degeneration of brain neurons	Sanchez-Garcia and Fernandez-Funez, 2018
S/D167	Mice expressing mouse PrP-D167S	High expression increased susceptibility to TSE. Moderate expression did not	Sigurdson et al., 2011
S/N174	X-ray crystallography on rabbit PrP-WT vs. rabbit PrP-S174N	Helix-capping domain form interactions of residues N171 and S174, eliminated by S174N	Khan et al., 2010
S/N174	Rabbit PrP-WT vs. rabbit PrP-S174N treated with urea	Rabbit PrP-S174N populated the beta-state, but rabbit PrP-WT did not	Khan et al., 2010
S/N174	Cell culture of mouse PrP-N174S	N174S was protective against TSE	Vorberg et al., 2003
S/N174	PMCA with recombinant rabbit PrP-S174N	S174S did not protect against TSE	Eraña et al., 2017
S/N174	PMCA with recombinant mouse PrP-N174S	N174S was protective against TSE	Eraña et al., 2017
S/N174	Fruit flies expressing rabbit PrP-S174N	S174N did not cause neurodegeneration	Sanchez-Garcia and Fernandez-Funez, 2018

To further test the *in vivo* protective activity of D159, S167, and S174, we recently generated transgenic flies expressing human PrP carrying these three variants: N159D, D167S, and N174S alone and in combinations. Human PrP is highly toxic in flies, making it an ideal model for testing putative protective residues (Fernandez-Funez et al., 2017). These studies will provide further evidence for the ability of these three key amino acids to suppress the toxicity of the highly toxic human PrP. As we wait for those experiments, it is important to consider the possibility that, based on the above results, single amino acid changes are unlikely to introduce substantial conformational changes in human PrP. Thus, it is likely that even subtle changes in the size of the side chains can impact the ability of the β2-α2 loop to interact closely with helix 3 and stabilize the CT3D domain. Therefore, double or triple mutations should be introduced in coordinated combinations, i.e., they need to come from the same animal to add the cooperative effect of multiple small changes. Alternatively, it is possible that we are still missing the key residues encoding PrP conformation and toxicity, particularly those encoding the high toxicity of human PrP. There are several areas of sequence and structure divergence between human PrP and rabbit, dog, and horse PrP in the CT3D domain (Figure 1A). It is likely that substitutions in the β2-α2 loop and helix 3 work cooperatively to increase the stability of this domain, but the distal helix 3 has received little attention in experimental studies thus far. For instance, the tammar wallaby carries A225,A226 in helix 3 instead of the common Y225,Y226 in human PrP and other animals, two substitutions that replace bulky tyrosines with the smaller alanines, possibly promoting closer contacts between helix 3 and the β2-α2 loop (Christen et al., 2009). However, little is known at this time about the susceptibility of wallabies to TSEs. Interestingly, rabbit, horse, and pig PrP carry relevant substitutions in these two positions that could contribute to the stability of the entire CT3D domain in combination with S167 and S174. We are currently examining the protective effect of introducing Y225A in distal helix 3 into human PrP, a first look at the role of distal helix 3 in the conformation of human PrP.

## Discussion

The natural variability in susceptibility to TSE and the exceptional resources to analyze PrP sequence and structure provide unique opportunities to decipher the code governing PrP misfolding, toxicity, and infectivity. The basic hypothesis is that amino acid changes between susceptible and resistant animals are responsible for the different conformational stability of PrP. Sequence alignments and structural studies identified three residues proposed to mediate the stability of dog (D159), horse (S167), and rabbit and pig (S174) PrP. The studies reviewed here show partial support for the protective activity of these three residues. Although differences between assays should be taken into consideration, the results discussed so far suggest that D159, S167, and S174 alone are insufficient to explain the different structural properties of highly resistant PrPs. Beyond these three residues, two systematic studies demonstrated the contribution of other resides to the differences between mouse and rabbit PrP while disagreeing on the role of S174 (Vorberg et al., 2003; Eraña et al., 2017). Thus, cracking the code of PrP toxicity requires expanding our focus to subtle amino acid differences *in combination* with D159, S167, and S174. Structural studies suggest that PrP stability is partially encoded in a rigid β2-α2 loop and in strong interactions between the loop and distal helix 3, resulting in a compact and stable CT3D domain.

The next efforts should be focused on defining the combinations of amino acids that achieve these structural goals. Generating double, triple, and multiple mutants is time consuming, particularly testing them in animal models. The structural data is crucial to define the best candidates to cooperatively stabilize the CT3D domain, which can be followed by *in silico* predictions of the impact of these combinations in molecular dynamics simulations. Remarkably, the main fold of the globular domain is not affected by point mutations, which instead appear to change the structure and dynamics of protein subdomains. This observation, combined with the small size of PrP, makes molecular dynamics simulations an ideal tool to systematically probe the effects of mutations on the conformational dynamics of PrP (van der Kamp and Daggett, 2011). The work by Daggett (Alonso et al., 2001; DeMarco and Daggett, 2004), Caflisch (Huang and Caflisch, 2015a, b), Parrinello (Caldarulo et al., 2017), and others, has shown that computer simulations can enrich experimental structural information by providing a description of the conformational landscape accessible to a mutant. In addition, provided appropriate sampling of the phase space, simulations can determine the thermodynamic stability of local conformations, the kinetics of their transitions, and validate these data against experimental observables (Caldarulo et al., 2017). Importantly, molecular dynamics simulations provide an atomistic description of the interactions that stabilize or destabilize a certain conformation, which allows to make predictions about the effect of mutations, and to rapidly test these predictions *in silico* (Huang and Caflisch, 2015a, b). Combinations with a significant impact on simulations can then be tested in transgenic animals, starting with fruit flies due to the fast and economic process of determining the toxicity of the mutants.

We still have extensive work to do to crack the complex code regulating PrP toxicity, which seems to involve subtle effects from multiple residues in the globular domain that, combined, result in vastly different morphotypes and phenotypes. Moreover, recent work focused on PrP glycosylation revealed two mechanisms for glycans to impact PrP aggregation, replication, and toxicity (Katorcha et al., 2014; Callender et al., 2020; Makarava et al., 2020). One is steric limitations due to the large size of the glycans. The second one can be even more important: glycans can be terminally sialylated, which adds a significant negative charge that prevents direct stacking of monomers but is compatible with a rotation (Baskakov et al., 2018). Thus, post-translational modifications add significant restrictions to the quaternary structures that misfolded PrP can explore. These restrictions are imposed on top of the tertiary conformations accessible based on the internal dynamics of several sub-domains. Overall, variations in both sequence and glycosylation limit and direct the generation of unique prion strains, which are defined by biochemical properties and phenotype (neurotropism and clinical symptoms). These considerations also help explain how different phenotypes can originate from the same sequence. Whereas there is clear experimental evidence that the PrP ordered domain adopts a single well-defined structure, the structure of the pathogenic misfolded monomers *and* aggregates may vary (Bessen and Marsh, 1994; Caughey et al., 1998; Safar et al., 1998; Peretz et al., 2002; Cobb and Surewicz, 2009). In other words, different strains can be associated to different structures of the aggregates. Different glycosylation patterns have also been associated to different phenotypes (Cobb and Surewicz, 2009), which have been proposed to arise from intermolecular contacts involving glycans in PrP^Sc^. In addition, it is plausible that sequences showing high conformational dynamics of the β2-α2 loop and high exposure of hydrophobic residues may be more likely to generate multiple structures of the aggregates and, in turn, multiple strains. In the nucleation-polymerization model (Jarrett and Lansbury, 1993), a PrP sequence characterized by a dynamic loop adopting many conformations may be more prone to provide the right template structure for a variety of aggregates’ structures (and strains), whereas a sequence with limited conformational polymorphism may be able to provide the template only for one aggregate’s structure, and thus originate only one strain. Ultimately, understanding the code mediating the phenotype – morphotype – phenotype relationship for PrP may guide the design of compounds that stabilize PrP and prevents disease progression. Ultimately, similar rules may apply to prion-like proteins (tau, α-synuclein, Amyloid-β42) responsible for highly prevalent neurodegenerative disorders with a significant impact in society: Alzheimer’s and Parkinson’s diseases.

## Author Contributions

PF-F and RM conceived the study. PF-F, RM, and AC conducted the research and wrote and revised the manuscript. All authors contributed to the article and approved the submitted version.

## Conflict of Interest

The authors declare that the research was conducted in the absence of any commercial or financial relationships that could be construed as a potential conflict of interest.
